# Ingestão de Ácidos Graxos na Prevenção Cardiovascular: A Incessante Busca pela Adequação

**DOI:** 10.36660/abc.20240208

**Published:** 2024-05-07

**Authors:** Elisa Maia dos Santos, Grazielle Vilas Bôas Huguenin

**Affiliations:** 1 Instituto Nacional de Cardiologia Departamento de Ensino e Pesquisa Rio de Janeiro RJ Brasil Departamento de Ensino e Pesquisa – Instituto Nacional de Cardiologia, Rio de Janeiro, RJ – Brasil; 2 Universidade Federal Fluminense Departamento de Nutrição e Dietética Niterói RJ Brasil Departamento de Nutrição e Dietética – Universidade Federal Fluminense, Niterói, RJ – Brasil

**Keywords:** Ingestão de Alimentos, Ácidos Graxos, Prevenção Secundária

As doenças cardiovasculares (DCV) são atualmente a principal causa de mortalidade em todo o mundo.^1^ Diversos fatores de risco contribuem para o seu desenvolvimento e dentre os modificáveis os principais estão relacionados a comportamentos de estilo de vida, como tabagismo, atividade física e dieta.^2^ Em indivíduos em prevenção cardiovascular secundária a promoção de um estilo de vida saudável apresenta-se como uma estratégia para redução da recorrência de eventos.^3 - 5^ Contudo, lacunas para adesão a um estilo de vida saudável na prevenção secundária podem estar relacionadas à baixa integração entre a mudança de comportamento e os modelos tradicionais de cuidado ambulatorial.^6^

A nutrição apresenta um papel central na prevenção primária e secundária das DCV, mas relativamente há pouco tempo os alimentos têm sido considerados como um tratamento, e não somente como um complemento à terapêutica médica e farmacológica estabelecida. A dieta, particularmente, tem um impacto significativo no gerenciamento dos principais de fatores de risco modificáveis para DCV como obesidade, dislipidemia, diabetes e hipertensão.^4^

Padrões alimentares cardioprotetores, como a dieta do Mediterrâneo, preconizam que a proporção da ingestão de diferentes ácidos graxos pode ser tão relevante quanto às quantidades totais do nutriente^7^ e na prevenção secundária apresentam efeitos superiores na redução de eventos quando comparados a dietas com redução de gorduras.^3^ Há muito se sugere que a gordura saturada é prejudicial à saúde cardiovascular, porém, uma metanálise^8^ sugeriu que a redução da gordura saturada não pareceu afetar a mortalidade total ou a mortalidade por DCV.

O equilíbrio correto do consumo de gordura é fundamental para a saúde cardiovascular, no entanto, tal como acontece com os tipos de carboidratos e proteínas, as fontes e quantidades dessas gorduras necessitam estar balanceadas.^9^

Gorduras trans e saturadas, encontradas principalmente em alimentos processados, frituras e produtos de origem animal, devem ser consumidas com moderação. Estas gorduras estão associadas a um aumento do risco de DCV e seu consumo deve ser desestimulado.^10^ Em contraste, as gorduras poliinsaturadas, encontradas em alimentos como óleos vegetais, peixes e sementes, demonstraram ter efeitos protetores contra DCV quando consumidas em quantidades adequadas.^11^

Maiores reduções nos eventos cardiovasculares incluindo doença coronariana foram observadas em estudos que substituíram a gordura saturada por gorduras poliinsaturadas quando comparadas com gorduras monoinsaturadas, carboidratos ou proteínas.^8 , 12^ Assim, parece que a redução da gordura saturada e a substituição por gordura insaturada promove maior benefício cardiovascular, não necessariamente através da redução do consumo de gordura saturada mas considerando que parte desse efeito se refere a fonte dessa gordura como por exemplo laticínios versus ultraprocessados.

Nos resultados do estudo a que se refere este editorial,^13^ os autores enfatizam a baixa adesão dos participantes às recomendações estabelecidas para o consumo de gorduras. Os autores observaram que nenhum participante aderiu a todas as recomendações de consumo de gorduras de forma simultânea e mais da metade deles não aderiu a nenhuma recomendação. A adesão exclusivamente à recomendação de AGS foi a mais prevalente. Algumas das hipóteses levantadas pelos autores foram o acesso limitado a informações, dificuldade de compreensão das orientações nutricionais além de baixa adesão influenciada por questões econômicas ou pela falta de acesso a alimentos frescos e minimamente processados.^13^

Neste contexto torna-se urgente a elaboração de estratégias para aumentar a adesão às recomendações nutricionais já estabelecidas pelas diretrizes. Promover educação alimentar e conscientização sobre a importância de escolhas alimentares saudáveis é essencial para incentivar mudanças positivas nos hábitos alimentares da população. Campanhas de saúde pública, consultas nutricionais e programas de educação nas escolas são formas eficazes de disseminar informações sobre nutrição e promover escolhas alimentares mais saudáveis.^14^

Com o objetivo de oferecer subsídios aos profissionais de saúde da Atenção Primária para que orientem a alimentação de indivíduos portadores de fatores de risco cardiovasculares, o Ministério da Saúde elaborou a Dieta Cardioprotetora Brasileira baseada em alimentos típicos brasileiros.^15^ Sua característica principal é o desdobramento das recomendações nutricionais das diretrizes em práticas alimentares condizentes com a realidade da população priorizando o fácil acesso aos alimentos e valorizando a cultura alimentar regional através de estratégias interativas e lúdicas oferecendo subsídios para que os indivíduos promovam melhores escolhas alimentares.

Diante da urgência em aumentar a adesão às recomendações nutricionais estabelecidas, é essencial que profissionais de saúde, autoridades governamentais e a sociedade como um todo trabalhem em conjunto para implementar políticas e programas eficazes que promovam a educação alimentar e conscientização, visando a prevenção e controle das DCV e a promoção da saúde cardiovascular da população.
