# Supplementary material for: Ingestão de Ácidos Graxos na Prevenção Cardiovascular: A Incessante Busca pela Adequação
Source: Arq Bras Cardiol. 2024 May 7;121(3):e20240208. [Article in Portuguese] doi: 10.36660/abc.20240208 (PMC11081198; doi:10.36660/abc.20240208)
Supplement: Translation

# Translation: Fatty Acids Intake in Cardiovascular Prevention: The Incessant Search for Adequacy

**DOI:** 10.36660/abc.20240208i

**Authors:** Elisa Maia dos Santos, Grazielle Vilas Bôas Huguenin

**Affiliations:** 1 Rio de Janeiro RJ Brazil Departamento de Ensino e Pesquisa – Instituto Nacional de Cardiologia, Rio de Janeiro, RJ – Brazil; 2 Niterói RJ Brazil Departamento de Nutrição e Dietética – Universidade Federal Fluminense, Niterói, RJ – Brazil

**Keywords:** Eating, Fatty Acids, Secondary Prevention

Cardiovascular diseases (CVD) are currently the leading cause of mortality worldwide.^1^Several risk factors contribute to its development and among the modifiable ones, the main ones are related to lifestyle behaviors, such as smoking, physical activity, and diet.^2^In individuals undergoing secondary cardiovascular prevention, promoting a healthy lifestyle is a strategy for reducing the recurrence of events.^3 - 5^However, gaps in adherence to a healthy lifestyle in secondary prevention may be related to the low integration between behavior change and traditional models of outpatient care.^6^

Nutrition plays a central role in the primary and secondary prevention of CVD, but relatively recently, food has been considered as a treatment, and not just as a complement to established medical and pharmacological therapy. Diet, in particular, has a significant impact on the management of the main modifiable risk factors for CVD such as obesity, dyslipidemia, diabetes, and hypertension.^4^

Cardioprotective dietary patterns, such as the Mediterranean diet, recommend that the proportion of intake of different fatty acids can be as relevant as the total amounts of the nutriente,^7^and in secondary prevention, they have superior effects in reducing events when compared to diets with fat reduction.^3^It has long been suggested that saturated fat is harmful to cardiovascular health, however, a meta-analysis^8^suggested that reducing saturated fat did not appear to affect total mortality or mortality from cardiovascular disease.

The correct balance of fat consumption is fundamental for cardiovascular health, however, as with the types of carbohydrates and proteins, the sources and quantities of these fats need to be balanced.^9^

Trans and saturated fats, found mainly in processed foods, fried foods, and animal products, should be consumed in moderation. These fats are associated with an increased risk of cardiovascular disease and their consumption should be discouraged.^10^In contrast, polyunsaturated fats, found in foods such as vegetable oils, fish, and seeds, have been shown to have protective effects against CVD when consumed in adequate amounts.^11^

Greater reductions in cardiovascular events including coronary heart disease were observed in studies that replaced saturated fat with polyunsaturated fats when compared with monounsaturated fats, carbohydrates, or proteins.^8 , 12^Thus, it seems that reducing saturated fat and replacing it with unsaturated fat promotes greater cardiovascular benefit, not necessarily through reducing the consumption of saturated fat but considering that part of this effect refers to the source of this fat, such as dairy products versus ultra-processed foods.

In the results of the study referred to in this editorial,^13^the authors emphasize the low adherence of participants to the recommendations established for fat consumption. The authors noted that none participant adhered to all fat consumption recommendations simultaneously and more than half of them did not adhere to any recommendation. Adherence exclusively to the AGS recommendation was the most prevalent. Some of the hypotheses raised by the authors were limited access to information, difficulty understanding nutritional guidelines in addition to low adherence influenced by economic issues or lack of access to fresh and minimally processed foods.^13^

In this context, it is urgent to develop strategies to increase adherence to nutritional recommendations already established by the guidelines. Promoting food education and awareness about the importance of healthy food choices is essential to encourage positive changes in the population’s eating habits. Public health campaigns, nutrition consultations, and school-based education programs are effective ways to disseminate nutrition information and promote healthier food choices.^14^

Intending to offer subsidies to Primary Care health professionals to guide the diet of individuals with cardiovascular risk factors, the Ministry of Health created the Brazilian Cardioprotective Diet based on typical Brazilian foods.^15^Its main characteristic is the breakdown of nutritional recommendations from the guidelines into dietary practices consistent with the population’s reality, prioritizing easy access to food and valuing regional food culture through interactive and playful strategies offering support for individuals to promote better food choices.

Given the urgency to increase adherence to established nutritional recommendations, it is essential that health professionals, government authorities, and society as a whole work together to implement effective policies and programs that promote food education and awareness, aiming to prevent and control diseases. CVD and the promotion of cardiovascular health in the population.
